# Pulsed lavage cleansing of osteochondral grafts depends on lavage duration, flow intensity, and graft storage condition

**DOI:** 10.1371/journal.pone.0176934

**Published:** 2017-05-02

**Authors:** Yang Sun, Weibo Jiang, Esther Cory, Jason P. Caffrey, Felix H. Hsu, Albert C. Chen, Jincheng Wang, Robert L. Sah, William D. Bugbee

**Affiliations:** 1 Division of Orthopaedic Surgery, the Second Hospital of Jilin University, Changchun, Jilin, China; 2 Department of Bioengineering, University of California San Diego, La Jolla, CA, United States of America; 3 Center for Musculoskeletal Research, Institute of Engineering in Medicine, University of California San Diego, La Jolla, CA, United States of America; 4 Division of Orthopaedic Surgery, Scripps Clinic, La Jolla, CA, United States of America; Mayo Clinic Minnesota, UNITED STATES

## Abstract

**Introduction:**

Osteochondral allograft (OCA) transplantation is generally effective for treating large cartilage lesions. Cleansing OCA subchondral bone to remove donor marrow elements is typically performed with pulsed lavage. However, the effects of clinical and experimental parameters on OCA marrow removal by pulsed lavage are unknown. The aim of the current study was to determine the effects on marrow cleansing in human osteochondral cores (OCs) of (1) lavage duration, (2) lavage flow intensity, and (3) OC sample type and storage condition.

**Methods:**

OCs were harvested from human femoral condyles and prepared to a clinical geometry (cylinder, diameter = 20 mm). The OCs were from discarded remnants of Allograft tissues (OCA) or osteoarthritis patients undergoing Total Knee Replacement (OCT). The experimental groups subjected to standard flow lavage for 45 seconds (430 mL of fluid) and 120 seconds (1,150 mL) were (1) OCT/FROZEN (stored at -80°C), (2) OCT/FRESH (stored at 4°C), and (3) OCA/FRESH. The OCA/FRESH group was subsequently lavaged at high flow for 45 seconds (660 mL) and 120 seconds (1,750 mL). Marrow cleansing was assessed grossly and by micro-computed tomography (μCT).

**Results:**

Gross and μCT images indicated that marrow cleansing progressed from the OC base toward the cartilage. Empty marrow volume fraction (EMa.V/Ma.V) increased between 0, 45, and 120 seconds of standard flow lavage, and varied between groups, being higher after FROZEN storage (86–92% after 45–120 seconds) than FRESH storage of either OCT or OCA samples (36% and 55% after 45 and 120 seconds, respectively). With a subsequent 120 seconds of high flow lavage, EMa.V/Ma.V of OCA/FRESH samples increased from 61% to 78%.

**Conclusions:**

The spatial and temporal pattern of marrow space clearance was consistent with gradual fluid-induced extrusion of marrow components. Pulsed lavage of OCAs with consistent time and flow intensity will help standardize marrow cleansing and may improve clinical outcomes.

## Introduction

Transplantation of an osteochondral allograft (OCA) is a generally reliable and highly effective treatment for large chondral or osteochondral lesions.[1–7] The major steps for acquisition and implant of an OCA are recovery, safety testing, and storage of a donor joint at 4°C, delivery of the donor graft to the operating room, preparation of the host recipient site, and preparation and implantation of the OCA.[8] A typical OCA is prepared as a cylinder, with diameter of 10–35 mm and subchondral bone (ScB) with thickness of 3–10 mm, although sometimes more, depending on the lesion depth and surgeon experience and preference.[8, 9] After implant, the post-operative rehabilitation involves protected weight-bearing for 4–12 weeks, and resumption of full activity at 4–6 months.[9] The failures that do occur often involve OCA subsidence with ScB collapse;[10–12] thus, methods to achieve more reliable and effective bone remodeling and graft incorporation are of interest.

Effective cleansing of the marrow portion of OCA potentially enhances graft incorporation. The marrow portion of bone allografts is antigenic,[13, 14] so that OCA can elicit an immune response after transplantation, particularly for massive grafts.[15, 16] While the induction of humoral immunity[17] by allografts increases with graft size, current methods of preparing and placing allografts do not lead to clinical rejection.[18] Cells in the ScB of fresh OCA stored at 4°C do not appear to survive the storage process.[19] The osseous portion of an osteochondral graft is remodeled gradually through creeping substitution,[20–22] whereby host cells repopulate and remodel the ScB.[22–26] Thus, pulsed lavage to remove marrow elements is recommended to minimize the immune response and enhance bone incorporation.[13, 27].

In several other clinical situations, the ability of pulsed lavage to cleanse bone is affected by the duration and intensity of lavage, as well as the status of the bone. To enhance cement fixation of joint prostheses, pulsed lavage removes marrow and facilitates the penetration of bone cement, with pulsed lavage being more effective than manual syringe bulb irrigation.[28–30] To enhance the effectiveness of osteoinduction by morselized allograft bone, adipose tissue is removed by pulsed lavage increasingly with irrigation duration and volume.[31] In massive osteochondral grafts for joint salvage after tumor excision, frozen storage[32, 33] leads to a decrease in marrow viscosity[34] that may enhance marrow removal by irrigation. We know of no previous studies on the extent of marrow removal from OCA ScB by pulsed lavage.

Micro-computed tomography (μCT) is useful for identifying and localizing a variety of tissue structures at high resolution and in three dimensions.[35] The trabecular structure of bone can be delineated by μCT because of its strong X-ray absorption in comparison to that by fluid[36] and by fat.[37] Contrast agents for μCT, such as Hexabrix, can help identify tissues that differ in adsorption of contrast, through either exclusion of contrast[38] or adsorption of contrast, including in empty fluid spaces.[39] Thus, μCT can be useful for evaluating the cleansing of the ScB of osteochondral grafts, by distinguishing trabecular bone from surrounding marrow, by localizing marrow components that excludes contrast, and by localizing fluid-filled voids after marrow cleansing.

The aims of the present study were to use μCT to assess the cleansing effects of lavage on osteochondral cylinders (OCs) of typical clinical geometry for different (i) lavage durations, (ii) lavage flow intensities, and (iii) sample type and storage conditions.

## Methods

### Human osteochondral samples

From July, 2013 to April, 2016, osteochondral fragments from the human femoral condyle were obtained as the discarded tissue fragments from 16 patients undergoing total knee replacement (TKR) for osteoarthritis and as the unused portions of donor tissue fragments for 4 patients undergoing OCA transplantation, all with IRB waiver because the samples were anonymized. TKR fragments were used after storage at -80°C (FROZEN) or at 4°C (FRESH). The storage condition for FRESH samples was 1–3 days in a mixture of 10% Dulbecco’s Modified Eagle’s Medium and 90% phosphate-buffered saline (PBS), supplemented with 100 U/mL penicillin, 100 μg/mL streptomycin, and 0.25 μg/mL fungizone. OCA donor fragments (JRFOrtho, Centennial, CO) were also stored at 4°C for the 20–24 days between donor death and surgical implantation, and then at 4°C for an additional 1–3 days, in the medium/PBS solution described above.

OC samples, with a geometry that is used clinically, were then prepared from the osteochondral fragments. OCs from allograft donor tissues are referred to as OCA, since they are identical to those used clinically. OCs from TKR specimens are referred to as OCT, and were inspected to ensure they had residual articular cartilage without marked erosion or exposed bone. OCA and OCT samples were prepared to a diameter of 20 mm with an allograft coring reamer (Arthrex, Naples, Florida). While being cored, an osteochondral fragment was submerged in PBS to dissipate heat. Then, the core was trimmed at the base with an oscillating saw (Arthrex) to leave a 3.5–6.5 mm length of bone.

### Pulsed lavage

In the various experiments described below, lavage for 45 or 120 seconds (s) was with PBS at 21°C. Lavage was performed using a commercial system, fitted with either a Standard Tip or a High Flow Tip (InterPulse Handpiece with Bone Cleaning Tip, Stryker, Kalamazoo, Michigan). With either tip, the OCT base was positioned against the inner surface of the dispensing funnel, 5 mm from the nozzle of the Standard Tip and 11 mm from that of the High Flow Tip (due to differing sizes of the outflow funnel), manually toggling the OC base to expose all of the surface to the fluid stream. The lavage flow rates were measured to be 9.6±0.2 mL/s with a Standard Tip and 14.6±0.1 mL/s with a High Flow Tip; thus, with the Standard Tip, the lavage fluid volumes were 430 mL after 45 s and 1150 mL after 120 s, and with the High Flow Tip, the volumes were 660 mL after 45 s and 1750 mL after 120 s. The lavage method and parameters (120 s and a High Flow Tip) are those used by one of the authors (WDB) in clinical practice.

### Study design

The effect of lavage on the gross appearance of OC was assessed. For this experiment, six OC samples were distributed amongst the three groups: OCT/FROZEN (69 year old male and 61 year old female); OCT/FRESH (84 year old male and 55 year old female); and OCA/FRESH (31 and 34 year old males). One OC sample of each group served as a negative control and was not lavaged. The other OC sample of each group was lavaged with PBS for 120 s using the Standard Tip configuration. The OCA/FRESH sample was subsequently lavaged with PBS for an additional 120 s using a High Flow Tip. Each OC sample was then bisected through the central vertical plane with a diamond-edge wafering blade attached to a low speed saw (IsoMet^®^, Buehler, Lake Bluff, Illinois). Gross photographs of one of the cut surfaces were taken for qualitative analysis of trabecular bone and marrow distribution.

The effects of lavage duration and flow intensity on the location and extent of OC cleansing were assessed using μCT. For this experiment, a total of 17 d = 20 mm OC samples were used (**Table 1**): OCT/FROZEN (n = 7 total, 4 male, 3 female, 65±13 years old, mean±SD); OCT/FRESH (n = 7 total, 2 male, 5 female, 65±14 years old); and OCA/FRESH (n = 3 male donors, 29±9 years old). OCs were alternately μCT scanned and lavaged in order to allow assessment of marrow removal. Samples were μCT scanned initially within PBS with proteinase inhibitors (1 mM phenylmethanesulfonyl fluoride, 2 mM disodium ethylenediamine tetraacetate, 5 mM benzamidine hydrochloride, and 10 mM N-ethylmaleimide, PI) (*scan a*), then equilibrated with 20% Hexabrix (Guerbet, Bloomington, IN) in PBS+PI and scanned in the Hexabrix-containing solution (*scan b*), lavaged with PBS at standard flow for a total of 45 s, then equilibrated with Hexabrix and scanned (*scan c*), lavaged with PBS at standard flow for a total of 120 s (75 s in addition to the first 45 s lavage), and then equilibrated with Hexabrix and scanned (*scan d*). OCA/FRESH samples were subsequently lavaged with a High Flow Tip for another 45 s, equilibrated with Hexabrix, imaged (*scan c*), lavaged additionally for a total of 120 s, equilibrated with Hexabrix, and imaged (*scan d*). Each incubation in Hexabrix-containing bathing solution was at 4°C and for 48–56 hr to ensure equilibration. Imaging was performed with a μCT scanner (Skyscan 1076, Bruker, Kontich, Belgium) at (18 μm)^3^ voxel resolution, applying an electric potential of 100 kVp and a current of 100 μA, using 0.038 mm copper + 0.05 mm aluminum filters. All scans were registered to *scan a* in order to enable comparison and analysis of the same VOI. All image processing and analysis was performed with CTAn and Dataviewer (Bruker).

**Table 1:** Groups and treatments.

Grp	source	storage	flow	lavage duration (s)	sample size (n)
**1**	OCT	FROZEN	std	45, 120*	7
**2**	OCT	FRESH	std	45, 120*	7
**3**	OCA	FRESH	std	45, 120*	3
**4**	OCA	FRESH	high	45^#^, 120^#^^,^*	3^#^

* indicates samples was subjected to repeated lavage for 120 s after lavage at standard (std) flow for 45 s.

# indicates samples were subjected to repeated (additional) lavage treatments at high flow for both 45 s and 120 s.

### Image processing

For each sample, *scan a* was processed to define a cylindrical volume of interest (VOI) containing most of the ScB, including trabecular bone (TB) and marrow volume (Ma.V). From *scan a*, bone was segmented by thresholding, using a gray scale value midway between the average gray scale values of bone and of marrow. The VOI was set to span axially from the basal bone surface to the subchondral plate, and radially outwards to a diameter of 19 mm (0.5 mm from the circumferential edge); this was achieved by creating a mask, beginning with the segmented bone, dilating, eroding, applying a shrink-wrap in 3D to close the trabecular space, and then applying a circumferential erosion in 2D.

All *scans*, *a-d*, were then analyzed within the VOI for empty marrow space (volume), EMa.V, relative to Ma.V. For each sample, the total volume of marrow space, Ma.V within the VOI, was defined by masking out the bone (determined in *scan a*) expanded by one voxel to account for partial volume effects. Within the marrow space, EMa.V was then determined as voxels with gray scale values and distribution similar to that of the Hexabrix-containing solution surrounding the sample using Excel (Microsoft, Redmond, WA). Empty marrow volume fraction was then calculated as EMa.V/Ma.V. To illustrate the bone and marrow structure of individual samples, vertical cross-section images from *scans a-d*, as well as masked images within the VOI, were displayed.

### Statistics

The effects of storage condition, lavage duration, and flow intensity on irrigation efficacy (EMa.V/Ma.V) were analyzed. Data is presented as mean ± SE. The EMa.V/Ma.V data were Arcsine transformed to improve normality before statistical testing. Statistical significance was set at α = 0.05. First, the effects of storage condition (OCT/FRESH, OCT/FROZEN, OCA/FRESH) and lavage duration (repeated measures at 0 s, 45 s, 120 s) were assessed by 2-way repeated measures ANOVA. When the effects of storage condition were significant, their effect was assessed at each lavage duration by 1-way ANOVA with post-hoc Tukey test. The effects on OCA/FRESH of flow (standard vs high) as well as lavage duration (45 s, 120 s) were assessed by 2-way repeated measures ANOVA.

## Results

### Effect of lavage on OC gross appearance

**Fig 1:**
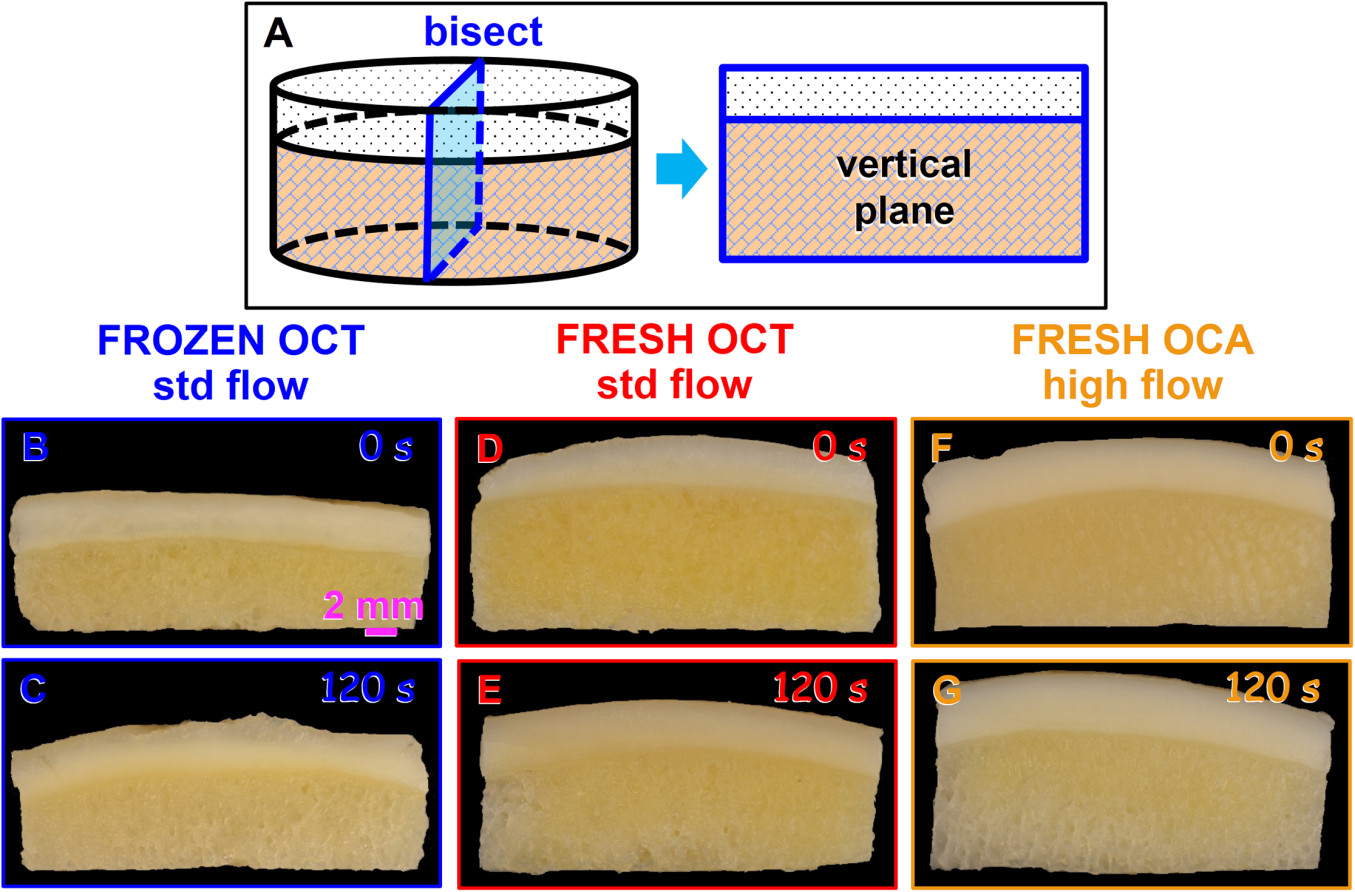
Representative gross images of OCs after different storage and lavage treatments. (**A**) Photos were taken of the vertical plane of bisected OC samples. (**B,C**) OCT/FROZEN samples, (**D,E**) OCT/FRESH samples, and (**F,G**) OCA/FRESH samples after irrigation for (**B,D,F**) 0 s or (**C,E**) 120 s with standard (std) flow, or (**G**) additional 120 s of high flow.

Qualitatively, overall OC cleansing efficacy appeared sensitive to irrigation for 120 s and storage condition. In gross photographs of OC bisected vertically, certain features were evident for OCT/FRESH, OCT/FROZEN, and OCA/FRESH sample types, and with irrigation intensity **(Fig 1)**. Without irrigation, the ScB of OC samples was yellow overall, with small ~0.5 mm wide areas that were light-yellow, consistent with marrow space, and thinner, irregular white structures, consistent with bone trabeculae. After 120 s of irrigation at regular or high flow, areas extending from the bottom edge were gray-white, while the central area remained yellow, particularly for the OCT/FROZEN sample. Thus, the macroscopic appearance of bisected samples indicated that lavage cleansing extended from the basal edge toward the center of the OC sample.

### Effects of lavage duration and flow intensity on the location and extent of cleansing of OC samples

**Fig 2:**
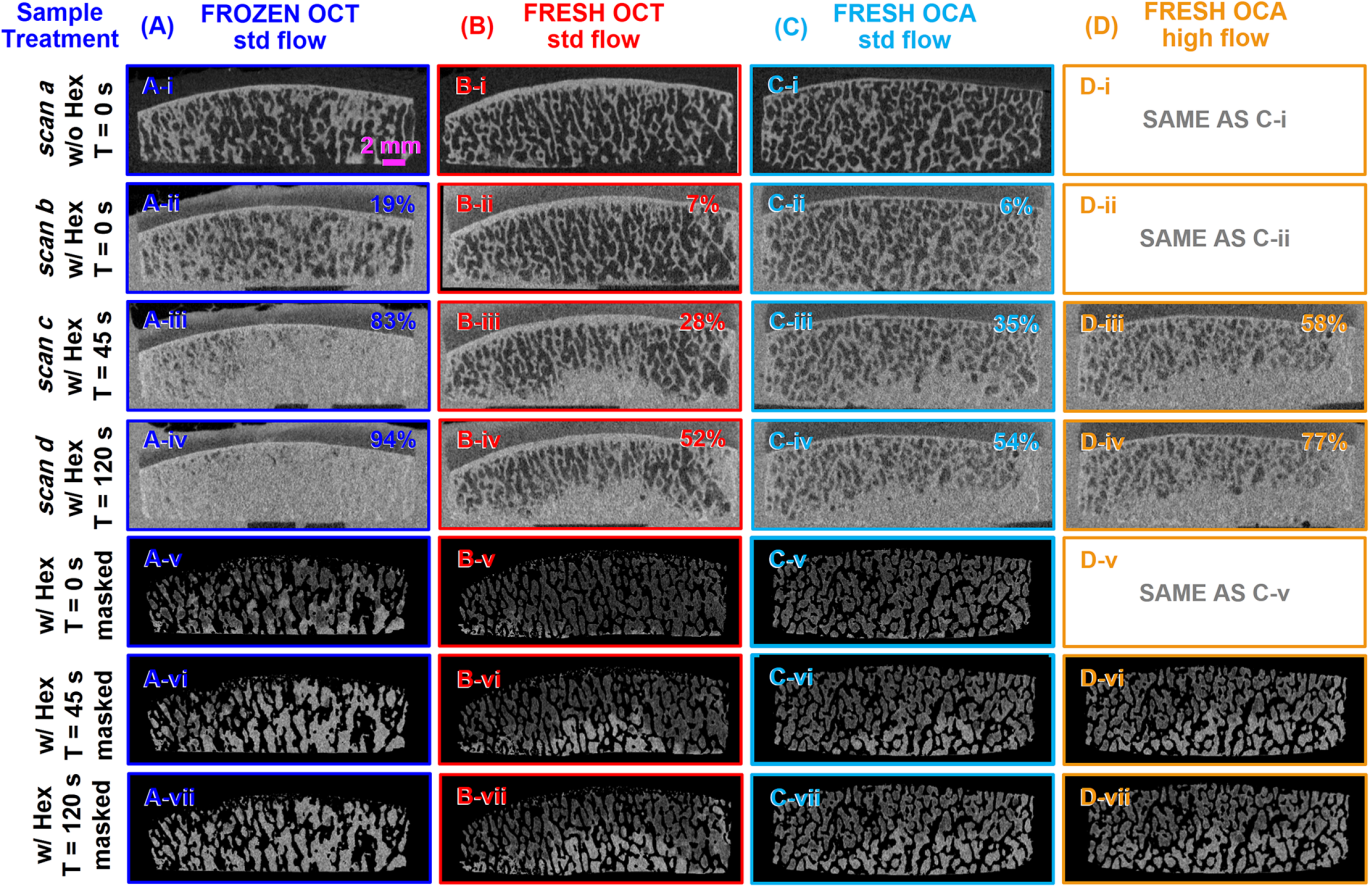
Representative μCT images of large contoured OCs after different storage and lavage treatments. Samples, (**A**) OCT/FROZEN, (**B**) OCT/FRESH, (**C, D**) OCA/FRESH, were irrigated for (**i, ii**) 0 s, (**iii**) 45 s, and (**iv**) 120 s with (**A, B, C**) standard (std) flow or (**D**) high flow. Samples were imaged (**i**) without contrast or with Hexabrix contrast (**ii**) before and (**iii, iv**) after irrigation. (**v, vi, vii**) Post-treatment images (**iii, iv, v**) were masked to remove trabeculae and surrounding tissues and fluid. All sequential images (**i-vii**) were registered.

Registered μCT images of OCT/FRESH, OCT/FROZEN, and OCA/FRESH samples showed spatial patterns after different durations and intensities of irrigation (**Fig 2**) that were consistent with the qualitative photos (**Fig 1**). In the ScB of *scan a* (**Fig 2-i**), before irrigation and without Hexabrix contrast, trabeculae were evident as thin irregular (~0.1–0.3 mm thick) bright stripes, with marrow evident as interspersed dark areas. Visible above the ScB was the subchondral plate, a bright horizontal strip (~0.2–0.5 mm thick). In the ScB of *scan b* (**Fig 2-ii**) and the corresponding image with the mineralized tissue masked out (**Fig 2-v**), before irrigation but with Hexabrix contrast, the trabeculae were slightly brighter, and the marrow regions were variably brighter. In the OCT/FRESH (**Fig 2B-ii,v**) and OCA/FRESH (**Fig 2C-ii,v**) samples, areas of marrow were dark indicating contrast was excluded, while in the OCT/FROZEN samples (**Fig 2A-ii,v**), marrow space was variably brighter, with some contrast infiltration. Thus, the image processing procedure inferred bone trabeculae from the bright pixels of *scan a* rather than attempting to infer trabeculae from *scan b*.

After lavage, the ScB regions of *scan c* (**Fig 2-iii,vi**) and *scan d* (**Fig 2-iv,vii**), taken with Hexabrix contrast, showed variable and localized increases in opacity, indicating marrow space that was infiltrated by contrast. With lavage duration increasing from 45 s (**Fig 2-iii,vi**) to 120 s (**Fig 2-iv,vii**), the area of increased contrast (brighter) extended from the base of the OC towards the top especially in the central region. In OCT/FROZEN samples, most of the ScB area was bright gray after 45 s (**Fig 2A-iii,vi**), with a slight increase after 120 s irrigation (**Fig 2A-iv,vii**), In contrast, in OCT/FRESH samples, the increase in gray area was less, after 45 s (**Fig 2B-iii,vi**) and 120 s (**Fig 2B-iv,vii**), and localized to the lateral and bottom edges. In OCA/FRESH samples, the effects of irrigation at standard flow (**Fig 2C-iii,iv,vi,vii**) appeared similar to effects on OCT/FRESH samples. After additional high flow lavage of OCA/FRESH samples, the gray area was increased slightly (**Fig 2D-iii,iv,vi,vii**).

**Fig 3:**
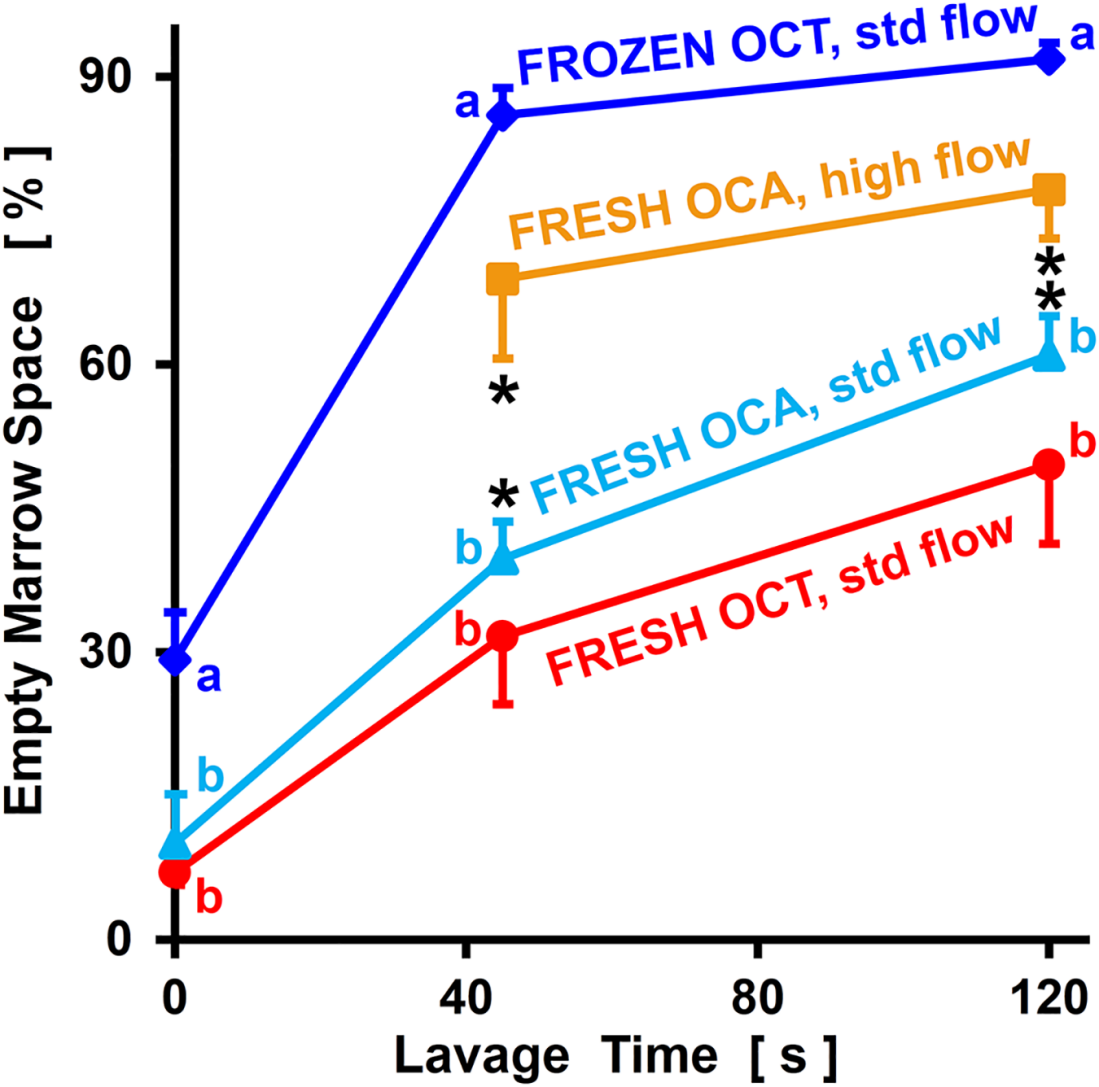
Percentage of empty marrow space of OCs after different storage and lavage treatments. Samples were OCT/FROZEN, OCT/FRESH, or OCA/FRESH, and subjected to standard (std) or high flow lavage for 0–120 s. Data are shown as mean ± SE, n = 3–7. Different letters (a, b) indicate experimental groups that are significantly different (p<0.05). * indicates the paired OCA/FRESH at standard flow and high flow groups are significantly different (p<0.05).

Quantitative image analysis confirmed that marrow cleansing at standard flow, as indicated by EMa.V/Ma.V, was affected by lavage duration (p<0.001) and sample type (p<0.001), with significant interaction (p<0.01, **Fig 3**). Considering all sample groups, EMa.V/Ma.V increased from 0 s to 45 s and also from 45 s to 120 s (each, p<0.001). At each time point (0 s, 45 s, and 120 s), EMa.V/Ma.V was not significantly different between OCT/FRESH and OCA/FRESH samples (p = 0.58–0.85), but was higher for OCT/FROZEN samples (each, p<0.05). For OCT/FRESH and OCA/FRESH samples, EMa.V/Ma.V increased from 9% at 0 s, to 36% at 45 s, and 55% at 120 s. For OCT/FROZEN samples, EMa.V/Ma.V was already 29% initially, and increased to 86–92% by 45–120 s.

Additional lavage of OCA/FRESH samples with high flow resulted in EMa.V/Ma.V being greater with high flow (p<0.05) and the long duration (p<0.05), without an interactive effect (p = 0.22). At 45 s of lavage, EMa.V/Ma.V increased from 40% after standard flow to 69% after high flow. At 120 s of lavage, EMa.V/Ma.V increased from 61% after standard flow to 78% after high flow.

## Discussion

These results demonstrate that OC marrow cleansing is sensitive to lavage duration, lavage flow intensity, and sample type (storage conditions). Lavage resulted in cleansing that started at the sample base, and progressed upwards especially in the central region (**Figs 1** and **2**). Marrow cleansing of each of the groups increased sequentially with standard flow lavage between 0 s, 45 s, and 120 s, and increased further for OCA/FRESH samples subjected to subsequent high flow lavage for 45 s and 120 s (**Figs 1–3**). OCT/FROZEN samples were cleansed more readily than OCT/FRESH and OCA/FRESH samples (**Figs 1–3**).

The design of the present study involved selection of a number of experimental parameters. Samples were both OCT, from TKR remnants, and OCA, from unused allograft remnants. Since OCAs are in high clinical demand, donor tissue was not readily available. In contrast, discarded fragments from TKR surgery are plentiful, and were screened so that OCT specimens were from a location with articular cartilage that was intact or only mildly eroded. In addition, a number of samples were tested repeatedly, at 0, 45, and 120 s of total lavage time, at standard flow and high flow, both to conserve samples and to increase the power of elucidating differences between time points. The high flow lavage was only applied to the OCA/FRESH group because the OCT/FROZEN group was highly cleansed even at 45 s lavage time, because of the clinical relevance of OCA/FRESH over OCT/FRESH, and because of the similarity between OCA/FRESH and OCT/FRESH under standard flow conditions. The effect of OCA/FRESH storage time was not assessed. All OC samples were prepared to a single clinically-relevant size, 20 mm diameter, to minimize effects of geometry. Lavage was applied using one manufacturer’s instrument, but whose flow rate was determined. These acquisition and usage processes thus provided sufficient sample numbers and also defined the clinical and experimental parameters in detail.

The present study utilized sequential μCT imaging, initially without contrast and then with Hexabrix contrast equilibration and measurement after lavage, to localize and quantify marrow cleansing as empty space between bone trabeculae. While fat, fluid (and hydrated tissues), and bone exhibit intrinsic differences in X-ray absorption, the relatively small difference between fat and fluid made it practical to use Hexabrix contrast to identify empty marrow space that was accessible, versus filled marrow space that was inaccessible. Since Hexabrix contrast obscured the boundary between trabeculae and marrow space, identification of bone trabeculae was performed using the initial non-contrast μCT scans. Thus, image processing of sequential μCT scans, without and with contrast, facilitated both localization and quantitation of marrow cleansing.

The present study provides important information on the extent of OCA cleansing, with certain similarities to, and differences from, results of previous studies of bone cleansing for other clinical scenarios. In studies of cancellous surface preparation for application and penetration of bone cement, pulsed lavage removed marrow to a depth ranging from 1.5 mm to >3 mm.[28–30] The volume of solution applied by pulsed lavage was important, with longer duration and higher fluid volume leading to increased cleansing;[31] conversely, even with five different commercial lavage systems, when a constant 500 mL of saline was used, cleansing occurred to generally similar depths.[28] Since the cleansing of samples in the present study did not occur in a constant planar fashion, extrapolation of the EMa.V/Ma.V to an effective thickness is not straightforward, but can be estimated by normalizing to the cross-sectional area and height of the specimen; such normalization gives effective cleansing depths averaging 1.4 mm after 45 s of standard flow for OCT/FRESH, 3.5 mm after 120 s of high flow for OCA/FRESH, and 3.8 mm after 120 s of standard flow for OCT/FROZEN.

The trends in extent of cleansing of different experimental groups (**Fig 3**) may reflect a number of factors. The more extensive cleansing of FROZEN relative to FRESH samples is consistent with the decreased viscosity of marrow after freeze-thaw.[34] Freeze-thaw disruption of marrow components, allowing for contrast penetration, would also explain the relatively high EMa.V/Ma.V in OCT/FROZEN samples compare to FRESH samples, even without lavage (i.e., at 0 s of lavage). The slight trend toward more irrigation-induced cleansing of OCA/FRESH samples compared to OCT/FRESH samples may reflect both practical and intrinsic tissue factors. A main practical factor that may have caused this trend was the 20–24 day storage of OCA/FRESH during graft processing; such storage may have caused some deterioration of the integrity of the marrow. An intrinsic factor that would have been expected to counter this trend is that OCT samples were from relatively old and female individuals, while OCA samples were from relatively young and male donors; this may have led to OCT samples being more osteoporotic than OCA samples, which may have made the former more susceptible to lavage. Finally, a neutral age-related factor may have been the transition from red marrow to yellow marrow, which occurs in the human femur by 24 years of age.[40] Even with such differences in sample populations, there was no marked difference in EMa.V/Ma.V between OCT/FRESH and OCA/FRESH samples without lavage. Thus, the present results also indicate that for translational laboratory studies on OC cleansing, OCT/FRESH samples created from femoral condyle remnants discarded during TKR surgery are a useful model system.

Further study would help to generalize conditions for optimal cleansing of OCA and an improved clinical outcome. From a biomechanical perspective, the variation in OCA size and structure would likely affect the extent of cleansing. From a biological perspective, the effects of cleansing on reducing immunogenicity and on enhancing biological incorporation may be similar. In addition, bone remodeling may be accelerated beneficially with more cleansing. On the other hand, the gradual nature of creeping substitution may also be effective, and depend on a region of marrow that is slowly remodeled. The present study delineates the location and extent of OCA cleansing with standardized lavage protocols, and provides foundation information for future research and, ultimately, to improve clinical outcome.

## Supporting information

S1 Table Controlled parameter and measured variable values for each experimental sample, as well as means, SE, and SD of each experimental group. (XLSX)
